# Complete Genome Sequence of *Herpesvirus anguillae* Strain HVA980811 Isolated in Chiayi, Taiwan

**DOI:** 10.1128/genomeA.01677-16

**Published:** 2017-02-23

**Authors:** Chiu-Ming Wen, Ping-Chung Liu, Fan-Hua Nan

**Affiliations:** a Department of Life Sciences, National University of Kaohsiung, Kaohsiung, Taiwan; b Department of Aquaculture, National Taiwan Ocean University, Keelung, Taiwan

## Abstract

*Herpesvirus anguillae* (HVA), also known as anguillid herpesvirus 1 (AngHV1), is one of the relevant viruses in wild and cultured anguillid eels. Here, the complete genome sequence of strain HVA980811, isolated in Chiayi, Taiwan, is reported. The genotype of the eel herpesviruses in Taiwan is supposed to be identical to the Japanese AngHV1.

## GENOME ANNOUNCEMENT

*Herpesvirus anguillae* (HVA), also known as anguillid herpesvirus 1 (AngHV1) and eel herpesvirus, was isolated first in Japan in 1985 from cultured moribund European eels (*Anguilla anguilla*) and Japanese eels (*Anguilla japonica*) (1). Complete genome sequence suggests that AngHV1 and the cyprinid herpesviruses are included within the family *Alloherpesviridae* (2). In Taiwan, eel herpesvirus Formosa (EHVF) and European eel herpesvirus (EEHV) were isolated from cultured diseased Japanese eels in the central regions and European eels in the northern regions, respectively (3, 4). Serological examination and partial sequence alignments proposed that both viruses are homologous to AngHV1 (4–6). Additionally, detection and isolation of AngHV1 have been reported from asymptomatic and diseased wild and cultured anguillid eels in Europe (7–14).

An eel herpesvirus strain, HVA980811, has a sequence (GenBank accession no. HQ992952) identical to the AngHV1 open reading frame 12 (ORF12) and was isolated from hemorrhaged Japanese eel farmed in Chiayi, south Taiwan, in 2009 (15). For genome sequencing, HVA980811 was propagated in EK-1 cells, and the virions were purified according to methods described previously (5). The viral genomic DNA was extracted by phenol extraction and delivered to Welgene Biotech (Taipei, Taiwan). The DNA was further extracted using a WelPrep DNA kit by Welgene and sonicated using a Misonix, Inc. 3000 sonicator for sizes ranging from 400 to 500 bp, and DNA sizing was performed with a bioanalyzer DNA 1000 chip (Agilent Technologies). One microgram of sonicated DNA was end repaired, A-tailed, and adaptor ligated, according to the Illumina TruSeq DNA preparation protocol, and ConDeTri was implemented for trimming (16). Cleaned and filtered nuclear reads were assembled *de novo* using ABySS (17). All obtained sequences were aligned and manually reassembled.

The HVA980811 genome comprises 249,071 bp, including a 10,714-bp terminal direct repeat. The nucleotide sequence is 99% identical to the genome sequence of AngHV1 (GenBank accession no. FJ940765.3) and contains 139 predicted protein-coding open reading frames (ORFs), as does AngHV1. Protein-protein BLAST showed that all the amino acid sequences are 100% identical to the AngHV1 counterparts, except ORF1. The result suggests that the genotype of the eel herpesviruses in Taiwan, including HVA980811, EHVF, and EEHV, is identical to that of the Japanese AngHV1.

### Accession number(s).

The complete genome sequence of anguillid herpesvirus 1 strain HVA980811 has been deposited in GenBank under the accession no. KX027736.

## References

[1] Sano M, Fukuda H, Sano T 1990 Isolation and characterization of a new herpesvirus from eel, p. 15–31. *In* Perkins TO, Cheng TC (ed), Pathology in marine science, vol 3. Academic Press, New York, NY.

[2] van Beurden SJ, Bossers A, Voorbergen-Laarman MHA, Haenen OLM, Peters S, Abma-Henkens MHC, Peeters BPH, Rottier PJM, Engelsma MY 2010 Complete genome sequence and taxonomic position of anguillid herpesvirus 1. J Gen Virol 91:880–887. doi:10.1099/vir.0.016261-0. 20016040

[3] Ueno Y, Kitao T, Chen SN, Aoki T, Kou GH 1992 Characterization of a herpes-like virus isolated from cultured Japanese eels in Taiwan. Fish Pathol 27:7–17. doi:10.3147/jsfp.27.7.

[4] Chang PH, Pan YH, Wu CM, Kuo ST, Chung HY 2002 Isolation and molecular characterization of herpesvirus from cultured European eels *Anguilla* *anguilla* in Taiwan. Dis Aquat Organ 50:111–118. 12180701 10.3354/dao050111

[5] Ueno Y, Shi J-W, Yoshida T, Kitao T, Sakai M, Chen S-N, Kou GH 1996 Biological and serological comparisons of eel herpesvirus in Formosa (EHVF) and *Herpesvirus anguillae* (HVA). J Appl Ichthyol 12:49–51. doi:10.1111/j.1439-0426.1996.tb00059.x.

[6] Shih HH, Hu CW, Wang CS 2003 Detection of *Herpesvirus anguillae* infection in eel using *in situ* hybridization. J Appl Ichthyol 19:99–103. doi:10.1046/j.1439-0426.2003.00353.x.

[7] Haenen OLMH, Dijkstra SGD, van Tulden PW, Davidse A, van Nieuwstadt AP, Wagenaar F, Wellenberg GJ 2002 Herpesvirus anguillae (HVA) isolations from disease outbreaks in cultured European eel, *Anguilla* *anguilla* in The Netherlands since 1996. Bull Eur Assoc Fish Pathol. 22:247–257.

[8] Lee NS, Kobayashi J, Miyazaki T 1999 Gill filament necrosis in farmed Japanese eels, *Anguilla* *japonica* (Temminck & Schlegel), infected with *herpesvirus* *anguillae*. J Fish Dis 22:457–463. doi:10.1046/j.1365-2761.1999.00197.x.

[9] Ginneken VJT van, Haenen O, Coldenhoff K, Willemze R, Antonissen E, van Tulden PW, Dijkstra S, Wagenaar F, van den Thillart G 2004 Presence of eel viruses in eel species from various geographic regions. Bull Eur Assoc Fish Pathol 24:268–271.

[10] Jakob E, Neuhaus H, Steinhagen D, Luckhardt B, Hanel R 2009 Monitoring of *herpesvirus* *anguillae* (HVA) infections in European eel, *Anguilla* *anguilla* (L.), in northern Germany. J Fish Dis 32:557–561. doi:10.1111/j.1365-2761.2009.01009.x. 19460086

[11] Bandín I, Souto S, Cutrín JM, López-Vázquez C, Olveira JG, Esteve C, Alcaide E, Dopazo CP 2014 Presence of viruses in wild eels *Anguilla* *anguilla* L, from the Albufera Lake (Spain). J Fish Dis 37:597–607. doi:10.1111/jfd.1392. 24846700

[12] van Beurden SJ, Engelsma MY, Roozenburg I, Voorbergen-Laarman MA, van Tulden PW, Kerkhoff S, van Nieuwstadt AP, Davidse A, Haenen OLM 2012 Viral diseases of wild and farmed European eel *Anguilla* *anguilla* with particular reference to the Netherlands. Dis Aquat Organ 101:69–86. doi:10.3354/dao02501. 23047193

[13] Varvarigos P, Vendramin N, Cappellozza E, Bovo G 2011 First confirmation of herpes virus anguillae (HVA) and infectious pancreatic necrosis (IPN) virus infecting European eels *Anguilla* *anguilla* farmed in Greece. Bull Eur Assoc Fish Pathol 31:101–111.

[14] Kempter J, Hofsoe P, Panicz R, Bergmann SM 2014 First detection of anguillid herpesvirus 1 (AngHV1) in European eel (*Anguilla* *anguilla*) and imported American eel (*Anguilla* *rostrata*) in Poland. Bull Eur Assoc Fish Pathol 34:87–94.

[15] Wen CM, Ku CC, Wang CS 2013 Viral susceptibility, transfection and growth of SPB—a fish neural progenitor cell line from the brain of snubnose pompano, *Trachinotus* *blochii* (Lacépède). J Fish Dis 36:657–667. doi:10.1111/jfd.12067. 23305502

[16] Smeds L, Künstner A 2011 ConDeTri—a content dependent read trimmer for Illumina data. PLoS One 6:e26314. doi:10.1371/journal.pone.0026314. 22039460 PMC3198461

[17] Simpson JT, Wong K, Jackman SD, Schein JE, Jones SJM, Birol I 2009 ABySS: a parallel assembler for short read sequence data. Genome Res 19:1117–1123. doi:10.1101/gr.089532.108. 19251739 PMC2694472

